# Electronic medical record alert increases HIV screening rates: the Foch hospital pilot POP-up project

**DOI:** 10.1186/s12913-022-08176-y

**Published:** 2022-06-16

**Authors:** Alexandre Vallée, Dimi Sveltlane, Julie Trichereau, Stéphane Neveu, Erwan Fourn, Catherine Majerholc, Philippe Lesprit, Laurence Mazaux, Seheno Harijaona Henintsoa, Grazyna Matejczuk, Marc Vasse, David Zucman

**Affiliations:** 1 Department of Epidemiology – Data – Biostatistics, Delegation of Clinical Research and Innovation, Foch Hospital, Suresnes, France; 2 Department of Internal Medicine, Réseau Ville Hôpital Val de Seine, Foch Hospital, Suresnes, France; 3 Département d’Informatique, Hôpital Foch, Suresnes, France; 4 Department of Hygiene and Infectious Disease, Foch Hospital, Suresnes, France; 5 Service de Biologie Clinique, Hôpital Foch, Suresnes, France

**Keywords:** Electronic medical record, HIV, HIV screening, Preventative care, Decision support

## Abstract

**Background:**

Despite significant national human immunodeficiency virus (HIV) screening activity, there are persistent delays in screening, and many missed diagnostic opportunities. To facilitate targeted screening, an electronic medical record (EMR) alert reminder was applied in the Foch hospital. Screening rates after implementation were reported.

**Methods:**

A prospective cohort analysis was performed in Foch Hospital between 24 April 2018 and 4 October 2019 among hospitalized patients born in high HIV prevalence countries and/or having social vulnerability criteria (universal health coverage). From the admissions software, when specific low health coverage was provided and/or high-prevalence country of birth was registered, an electronic alert (EMR alert) appeared on the ward where the patient was hospitalized. The EMR alert database was examined for HIV screening and activity responses from each service of the Hospital.

**Results:**

Eight thousand one hundred eighty-one alerts were recovered during the period for 1448 patients. 27 services used the EMR alert. Most of the alerts were directly closed (74.4%), 14.5% of the alerts were closed due to doctors declaring that they did not have time to respond. 297 (3.6%) of the 8181 alerts resulted in a prescription of HIV serology corresponding for 20.5% of the patients.

**Conclusion:**

EMR alert can help to increase the rate of HIV screening in hospital care practice. Through this EMR alert system, HIV screening can be implemented as a common practice like any other medical alternative. Future research should examine the factors influencing physicians’ attitudes to this alert system to improve the HIV screening rate.

## Introduction

In 2018, in France, about 25,000 people living with human immunodeficiency virus (HIV) were unaware of their HIV status. Despite significant national screening activity, there are persistent delays in screening, and many missed diagnostic opportunities. In 2018, an estimated 6800 people discovered their HIV-positive status. This number has been stable since 2007 [1]. The most common reason for testing is the presence of clinical symptoms.

Screening activity (number of serologies) is important in France, however, the number of late discoveries remains elevated [1]. Further research is therefore needed to improve targeting techniques for people living with HIV who are unaware of their HIV status. There are several barriers to HIV screening, such as thinking not to be at risk of acquiring HIV and not discussing risk-taking issues with the physician. For health professionals, the difficulty of speaking about HIV, avoidance of the subject of HIV, but also lack of training both to propose and do the test [2]. Similar studies in Europe and The United States have confirmed this finding [3–5].

Significant challenges remain in implementing published guidelines [6]. In France, it is recommended that at least one HIV test be offered to the general population, when using care and more frequently for at-risk populations.

There is no organized HIV screening strategy during hospitalization. HIV screening during hospitalization is rarely carried out except in certain services (maternity, internal medicine, and haemodialysis) while migrants and people living in precarious situations can be hospitalized in all departments of the hospital. On the other hand, patients often believe that they have had a routine HIV test done during their hospitalization whatever the pathology leading to hospital admission.

Moreover, the initial HIV serology prescription was very heterogeneous in our hospital. Before the implementation of this study, HIV screening was very poor in surgical departments of our hospital, which had led to several deaths, in particular cases of cerebral toxoplasmosis mistaken for cerebral metastases due to the lack of knwoledge of HIV infection in hospitalized patients. On the other hand, many services had a significant or even systematic use of screening tests (e.g. haemodialysis, maternity) representing a high number of tests. In parallel, the use of prescription alerts was implemented several years ago for multiresistant bacteria to antibiotics and emerging bacteria highly resistant to antibiotic screening and practices for isolating and carrier patients.

As a result, there are many missed opportunities for HIV screening in a hospital. Physicians and hospitals are increasingly being evaluated based on their adherence to screening recommendations. Electronic medical report alerts (EMR alert) have become a commonly used method to encourage physicians to use screening. Although many physicians have expressed concern about “alert fatigue,” the literature suggests that EMR improves screening rates for many conditions, including breast cancer, osteoporosis, abdominal aortic aneurysms, hepatitis C and obesity [7–11]. Despite the latest studies showing positive effects for HIV screening [12], EMR alert for HIV screening data remain uncertain. Indeed, several studies have shown an increase in HIV screening with EMR alert in association with other interventions [13–15], while other studies show on the contrary no effect of these EMR alerts [16].

Thus, the objective of our study was to assess the impact of the EMR alert reminder on rates of screening for HIV in a hospital care practice.

## Method

Foch hospital is a non-profit medical-surgical hospital located in France, access to which is open to the entire population of the area. Of the 44,000 annual hospitalizations, approximately 2000 vulnerable/precarious hospitalized patients (having state medical aid (AME) / universal health coverage (CMU) or complementary universal health coverage (CMUc)) have been identified. HIV screening activity has proven to be non-existent (< 100 tests/year) in the various surgical departments (thoracic, visceral, urology, neurosurgery, etc.) even though these departments receive as many disadvantaged patients as the medical departments. Throughout the hospital, about 2200 HIV serology tests are carried out per year (mandatory screenings carried out by the maternity department have been excluded). There are only 200 serology tests prescribed by the Emergency Department per year for more than 40,000 emergency visits. This situation is probably not specific to Foch hospital. Faced with these extremely low screening figures, it became necessary to set up a “POP-Up” electronic alert within the Foch hospital to raise the awareness of medical personnel and improve screening practices. This innovative research was a first in France.

Thus, a prospective cohort analysis, non-randomized and monocentric study was performed for all patients hospitalized in Foch Hospital between 24 April 2018 and 04 October 2019. The study was approved by the Foch IRB: 2016-A01194–47.

The major benefit of this research is to try to improve HIV screening practices among vulnerable and disadvantaged hospitalized patients at Foch Hospital.

The main objective of this study was to investigate the practice of hospital doctors in Foch hospital following the implementation of an EMR alert to encourage targeted screening for HIV infection according to specific socio-demographic criteria.

Patients were included if aged over 18 years, had AME, CMU or CMUc or were born in high HIV prevalence countries such as: sub-Saharan Africa, the West Indies, South America, Asia, Eastern Europe [17, 18]. In France, migrants, largely from sub-Saharan Africa, represent a significant proportion of HIV cases (38% in 2015). This population is mainly covered by AME/CMU. They represent 40% of undiagnosed HIV-infected individuals [19].

Exclusion criteria were people with engaged vital prognostic (unable to consent due to alteration of conscience) at the time of hospitalization, those not having given consent, and people already knowing their HIV status.

For patients: age, gender, health coverage, country of birth were recovered.

### EMR alert characteristics

To avoid the “fatigue reminder” effect [20] the window will not be blocking, ticking an item does not generate another window. Only one window will appear with all the necessary information. This simplicity proved effective in the study of Federman et al. [21].

From the “AXYA” admissions software, as soon as the type of AME or CMU health coverage are provided and/or the high-prevalence country of birth were registered, an electronic alert (a bridge between the admission software and the medical prescription software “OMNIPRO”) appears on the ward where the patient is hospitalized (computerized medical record).

The Computer Science Department of Foch hospital will extract using this software:

For the patient:

- Anonymized socio-demographic data of hospitalized patients: initials (1st letter of surname and 1st letter of first name), age, gender, country of birth, type of health cover (AME or CMU), serology performed (date and results).
- The name of the prescribing doctor and the service,
- The name of the hospitalization service.

For the doctor:

- The name of the service,
- The opening date of the POP-UP,
- The answers to the POP-UP questions described in paragraph 3.2.1.1.

Retrospective data for the last 12 months of patients hospitalized at Foch Hospital (no identification of patients will be extracted): number of hospitalizations, number of serologies performed, number of patients with socio-demographic criteria justifying HIV screening (medical coverage and country of birth).

In the POP-Up window physicians see:

1 - The current summary of recommendations for HIV screening.

2- Six checkboxes:

- Prescription of HIV serology (with brief note).
- No time to respond to the alert.
- Patient who has already had a serology dating from less than 3 months
- Patient followed for HIV infection.
- Patient who refused the test.
- Clinical condition of the patient not allowing to obtain his non-opposition.

This alert can only close if it is informed. If the alert was directly closed or the “no time to respond to the alert” was clicked, the alert was repeated every day during the patient hospitalization stay.

### Statistical analysis

Characteristics of the study population were described as absolute numerical values and proportions for categorical variables.

## Results

In total, 8181 alerts were recovered during the period for 1448 patients. 27 hospitalization units used the EMR alert. Most of the alerts were directly closed (74.4%), 14.5% of the alerts were closed due to clinicians declaring that they had no time to respond. 297 (3.6%) of the 8181 alerts resulted in a prescription of HIV serology but corresponding to 20.5% of the patients (Table 1). For 523 patients (36.1%) the EMR alert was unique since 23.9% of the alerts resulted in prescribed HIV serology (Table 1). For the remaining 925 when the EMR alert was repeated the median (25th to 75th percentile) of alerts was 6 [3 -10] and resulted in 18.6% of patients who were prescribed an HIV serology (Table 1).

**Table 1:** Distribution of medical responses to POP-UP alerts

	Overall responses (1.448 patients, 8.181 alerts)		Responses for MD of patients with only one hospitalization (523 patients)		Last response for MD of patients with multiple hospitalizations (925 patients)	
	n	%	n	%	n	%
0-Window directly closed	6087	74.4	118	22.56	513	55.46
1-Prescription of HIV serology	297	3.63	125	23.9	172	18.59
2-No time to respond to the alert	1188	14.52	29	5.54	81	8.76
3-Patient who has already had a serology less than 3 months old	307	3.75	196	37.48	110	11.89
4-Patient followed for HIV infection	65	0.79	49	9.37	15	1.62
5-Patient who refused the test	29	0.35	5	0.96	24	2.59
6-Clinical condition of the patient not allowing to obtain his non-opposition	208	2.54	1	0.19	10	1.08

Among the 1448 patients, 51.9% were male, 86.6% were CMU and 69.7% of them came from Africa. The mean age of patients was 55.9 [18–91] years (Table 2).

**Table 2:** Characteristics of the study population of patients

Parameters	n	%
Sex		
*Female*	697	48.1
*Male*	751	51.9
Health coverage		
*AME*	69	11.0
*CMU*	543	86.6
*SS*	15	2.4
*Missing data*	824	
Alert origin		
*Birth country*	836	57.7
*Social security*	484	33.4
*Both*	128	8.8
Geographic region		
*South America*	15	1.56
*Asia*	132	13.69
*Africa*	672	69.71
*Eastern Europe countries*	65	6.74
*Guyane*	8	0.83
*Haiti*	72	7.47
Age of patients	n	%
*≤ 30 years*	73	5.07
*]30–40 years]*	192	13.33
*]40–50 years]*	244	16.94
*]50–60 years]*	305	21.18
*]60–70 years]*	392	27.22
*> 70 years*	234	16.26

state medical aid (AME)

universal health coverage (CMU)

SS: social security

The first four services prescribing HIV serology with the EMR alert were nephrology (18.2% of total), neurology (16.2% of total), internal medicine (14.1% of total) and digestive surgery (10.4% of total) (Table 3). On the 297 HIV serologies, 182 gave a result (61.3% [57.7–68.9] of the total). 98.9% of the serologies were negative, while 2 were positive (1.1% [0.2–4.3]. One of the positive HIV tests was found in Cardiology, and the other in the Nephrology department, the triggering factor being social vulnerability for one and high prevalence country of birth for the other. One of the two patients admitted having already had an HIV positive test done. The newly discovered patient was linked to care during his hospitalization. Nephrology (40.6%), digestive surgery (40.3%) and neurology (34.5) remained the first services with a higher HIV serology prescription rate based on the number of hospitalizations (Table 3).

**Table 3:** Number of POP-UP alerts among number of hospitalizations for each service of the Hospital

Service	POP-UP alerts	hospitalizations	HIV serology prescriptions	% HIV prescriptions/hospitalizations	HIV serology made
Neurosurgery	956	137	16	11.7	10
Urology	765	148	9	6.1	3
Cardiology	699	133	23	17.3	15
Diabetology	657	48	12	25.0	10
Neurology	622	139	48	34.5	32
Thoracic surgery	570	68	8	11.8	3
Nephrology	553	133	54	40.6	28
Oncology	534	84	10	11.9	4
Internal medicine	452	156	42	26.9	29
Pneumology	440	113	20	17.7	10
Obstetric	425	75	2	2.7	0
Urgency	289	30	7	23.3	4
Digestive surgery	255	77	31	40.3	24
Psychiatry	243	20	3	15.0	3
Geriatrics	222	13	1	7.7	1
Otorhinolaryngology	211	30	2	6.7	1
Gynecology	125	25	0	0.0	0
Vascular surgery	92	12	4	33.3	3
Anesthesiology	38	22	3	13.6	2
Ambulatory surgery	10	10	0	0.0	0
Others	23	19	2	10.5	0

## Discussion

The purpose of this study was to assess the impact of the EMR alert on targeted HIV screening rates in hospital care practice. The results of the study showed that the EMR alert led to a prescription of HIV serology in 20.5% of the targeted population. Previous studies have shown that EMR was associated with a reduction in the number of patients who have never been screened for HIV [12–14]. Similarly, an Indian study showed that the introduction of an electronic recall for 1 year was associated with higher rates of HIV screening [15]. However, it appeared that these encouraging results would only be effective in the event of systematic recalls [16].

Overall, our results are complements to a growing number of findings supporting the use of an EMR alert, even for traditionally stigmatizing conditions such as HIV infection. However, only 61% of the prescribed serologies were carried out. The prescription was unfortunately only rarely followed up in service teams, one possible explanation could be patient refusal or lack of time by healthcare teams for non-priority care faced with the urgency of support.

EMR alert is easily usable by health professionals and consists of a simple prescription reminder to help health professionals to better screen precarious populations in the face of the national and international public health challenge that is HIV screening. Even if only one test was positive, the large number of tests carried out may show the need for such devices to allow better management of patients at risk. In the United-States, the use of this tool has been strongly encouraged by health authorities to avoid variability in practices but also medical errors in the management of HIV or other pathologies [22–24]. The introduction of the EMR alert is mainly accompanied by improved practices and often reduced costs [25]. EMR is not intended to replace the clinician’s judgment, but rather to provide a tool that helps health care teams manage effectively. Indeed, this tool allows them to have the latest recommendations of the experts and thus permits better clinical decisions. EMRs have proved effective in improving the care of people living with HIV. This improves HIV screening and that for other sexually transmitted infections [26–30]. An American team from Ohio [31] implemented an EMR system from the computerized patient record from July to December 2009. After its implementation there were four times as many HIV serologies prescribed. This alert was a reminder of HIV screening recommendations and sent an HIV screening order. This same experiment was done by a New York team, the implementation of the EMR allowed a marked increase in the number of serologies: 5.4% versus 8.7% [32].

Automatic recall would be particularly effective in increasing screening rates in patients with low baseline screening rates. Older adults have been identified as a population that has not met its screening goals. HIV screening among people aged 50–64 years increased slightly after recommended universal screening in 2006, and then decreased again over time to a prevalence of only 3.7% in 2010 [33].

A recent study showed that EMR significantly improved the use of screening, particularly in patients aged 46 to 65 years [12]. Low screening rates in older adults may reflect the beliefs of practitioners in a low epidemiological risk of HIV in middle-aged and older populations [34]. Although, this discrepancy supports the need for universal HIV screening that was recommended in 2009 in France [35], this strategy has not been implemented. Targeted screening appears more feasible and acceptable by health care professionals. However, the direct refusal of patients and the risk of stigmatization despite the goodwill of practitioners cannot be excluded and this fear remains a barrier for opt-in strategies.

The frequency of patients’ hospitalizations was not associated with the likelihood of receiving an HIV screening, with or without the EMR [12]. While it may be thought that more frequent visits would result in higher rates of HIV screening through numerous EMR alerts, it is possible that patients who are seen more frequently came for more episodic visits to solve acute problems, during which a prevention policy would not be the primary objective. Indeed, one might think that during these short hospitalizations, doctors would pay less attention to an EMR alert for these patients concerned.

Moreover, the implementation of this type of alert could be mainly effective if the participation rates of each service was increased by better communication tools. The main barrier for installation of such a POP-Up alert in other hospitals could be the specificity of the computer service systems which are different in each establishment, and which require ‘tailor-made’ tools. In future studies, it would be useful to collect data on the reason for the visit to better understand this finding. Although the results of this project are strongly positive, it should be noted that doctors’ overall adherence to this device remains low, with 74.4% of alerts being directly closed by doctors. This suggests that practices may need to consider alternative strategies to achieve screening goals. An alternative to “passive” EMR alert is “active” recalls [13]. In addition to an EMR alert, organizational factors may be needed to improve screening.

### Limitations

One of the strengths of our study is that it was conducted without any further intervention, including training of physicians in these screening issues [14]. All the hospitalization departments of the hospital participated in the study. However, it is important to note that in everyday life, HIV screening is usually proposed based on patient risk factors (risky sexual behaviour or intravenous drug use) independently of socioeconomic vulnerability criteria. The results could be more important in geographical areas with high-risk populations, while our population has only remained focused on socially vulnerable populations and patients born in high prevalence countries whatever their risk factors [14]. Our study must be considered with several limitations. A patient with a previous HIV diagnosis was included, the physician who followed the POP-Up request did not know this patient, and this could be added as a limitation of our study. Another limitation of our study was that we did not compare rates of HIV testing during the study period to rates of testing before implementing the alert, so it is difficult to assess the effect of the EMR alert. No information was added for specific higher testing situations, like the nephrology service which had a high screening rate for patients with renal insufficiency who potentially may require haemodialysis. Our data collection could not treat HIV screening outside our health care system. Therefore, our screening rate is likely to underestimate the actual number of patients who have been screened. Moreover, no screening rates during the period were reported in our hospital. This cannot us allowed a comparison between rates of HIV screening in the hospital and with the EMR alert. We did not investigate the “human” factors that are the main barriers to the use of these EMRs. A final shortcoming is the relatively short period of use of this recall system, about eighteen months. Therefore, it is not clear whether this EMR alert will have long-term effects.

## Conclusion

This study demonstrated that an EMR alert can help to increase the rate of HIV screening in hospital care practice. The progress is mainly qualitative in our study, in the departments which never prescribed serology (e.g., neurosurgery) which have started to do so thanks to the EMR alert. The services that were strong prescribers remained so and as their volume of prescriptions was high, it was difficult to show an increase in the mass of prescriptions. Through this EMR alert system, HIV screening can be implemented as a common practice like any other medical alternative. Physicians can also benefit from ongoing training on who to screen and how to avoid stigma in the face of HIV screening. Thus, a comprehensive approach combining EMR alert and medical training could improve HIV screening. Significant opportunities for improvement remain. Future research should examine the factors influencing physicians’ attitudes to this alert system but also other ways to improve this system and to improve the rate of HIV screening.

## Data Availability

The datasets generated and/or analysed during the current study are not publicly available due to the French law and but are available from the corresponding author on reasonable request.
